# Retraction: Modeling cardiac dysfunction following traumatic hemorrhage injury: impact on myocardial integrity

**DOI:** 10.3389/fimmu.2024.1548141

**Published:** 2025-01-07

**Authors:** Frontiers Editorial Office

Following publication, concerns were raised regarding the integrity of the images in the published figures. Specifically, unexpected similarity was detected between images presented in Figures 4 and 5. An investigation was conducted in accordance with Frontiers’ policies. The authors provided raw data for assessment, but the concerns could not be fully resolved. As a result, the data and conclusions of the article have been deemed unreliable and the article has been retracted.

This retraction was approved by the Chief Editors of Frontiers in Immunology and the Chief Executive Editor of Frontiers. The authors received a communication regarding the retraction and have agreed to this retraction. This communication has been recorded by the publisher.

Frontiers would like to thank Aneurus Inconstans for contacting the journal regarding the published article.

